# Editorial: Radiation therapy using MRI-Linac - the right way to start: a guide for physicians and physicists

**DOI:** 10.3389/fonc.2023.1258257

**Published:** 2023-08-09

**Authors:** Enis Özyar, Merav A. Ben-David, Frank Lagerwaard

**Affiliations:** ^1^ Radiation Oncology Department, Acıbadem University, Istanbul, Türkiye; ^2^ Oncology Department, Assuta Medical Center, Tel Aviv, Israel; ^3^ Ben-Gurion University of the Negev, Beer-Sheva, Israel; ^4^ Radiation Oncology Department, VU Amsterdam, Amsterdam, Netherlands

**Keywords:** MR guided radiotherapy, sterotactic radiotherapy, online adaptive, reoptimization, MR imaging

Radiotherapy iss an important method of local-regional treatment of malignancies and has witnessed impressive advancements in recent years. The main aim of radiotherapy is to increase the outcome of patients while minimizing side effects. The emergence of a new cutting-edge hybrid technology - Magnetic Resonance Image-guided Linear Accelerators (MR-Linac) - is a revolutionary breakthrough technology in the field. This new technology combines real-time MR imaging of “the anatomy of the day”, prediction of the dose distribution, online adaptive optimization of the plan if needed, and continuous automatic cine-MR tracking of the target. As this technology has a significantly different workflow compared to conventional radiotherapy, it is crucial to understand the fundamentals of utilizing MR-Linac to reach the goal to obtain optimal patient outcomes. The following topics we would like to emphasize are the major important components of MR-Linac technology;

## 1 Exploiting the power of MR imaging during online adaptive radiotherapy

One of the key influences of MR-Linac is the ability to integrate high-quality real-time MR imaging into the MR-guided radiotherapy workflow. Physicians must notice the implication of this feature to visualize the exact location of tumors and critical structures immediately before the treatment, recontouring or editing tumors and neighboring normal organs and structures if needed, and tracking the tumors continuously during beam-on and stop the treatment automatically if the target is out of the boundaries.

## 2 Collaborative approach of the treatment team

The successful implementation of a new MR-Linac workflow requires close teamwork between physicians, Radiotherapy Technologists (RTTs), and Medical Physicists. Each team member has a valuable role in the different steps of the workflow. This collaborative approach aims to synergistically exploit the capabilities of MR-Linac (1).

## 3 Online adaptive radiotherapy: optimizing precision and personalization

Members of the team should collaborate to develop optimal personalized treatment plans based on the “anatomy of the day” imaging where needed. A recent analysis of 50 patients with localized prostate cancer who were treated with ultra-hypofractionation using MRgRT in a total of 250 fractions has shown that in 76% (190/250 fractions) of fractions, reoptimizisation is needed due to various reasons (2).

## 4 Continuous monitoring of the target during beam on

This new technology enables the real-time tracking of the tumors, ensuring the preplanned optimal target dose.

The goal of this Research Topic is to collect and summarise the growing knowledge from institutions using online MR-guided radiotherapy. To share the obstacles, solutions, learning curves, and innovations of this new treatment modality. Eleven top-notch manuscripts are published in this new Research Topicof Frontiers in Oncology – Radiation Oncology Journal.

The first manuscript of this Research Topic aimed to evaluate the geometrical differences and metabolic parameters (FDG-PET, DWI-MRI) as a tool for an individualized definition of the volume in need of dose escalation for squamous cell esophageal cancer (Li et al.). Second manuscript reports the dosimetric benefits of daily adaptation of SMART and the first clinical results in pancreatic tumors in 30 patients (Michalet et al.). Third manuscript assesses the quality of a new diffusion-weighted imaging (DWI) sequence implemented on an MR-Linac MRIdian system, evaluating and optimizing the acquisition parameters to explore the possibility of clinically implementing a DWI acquisition protocol in a 0.35-T MR-Linac (Nardini et al.). The fourth manuscript reported the use of MRgRT for pediatric patients over four years and describes important considerations in the selection and application of this technology in children (Hall et al.). The fifth manuscript quantitatively characterizes the dosimetric effects of long on-couch time in prostate cancer patients treatment (Gao et al.). Sixth manuscript reports the workflow and initial clinical experience of high-grade glioma radiotherapy on the 1.5 T MR-Linac (MRL), with a focus on the temporal variations of the tumor and feasibility of multi-parametric image (mpMRI) acquisition during routine treatment workflow. (Tseng et al.) The seventh manuscript documented the critical steps needed for the appropriate delivery of MRgART for lung tumors safely and effectively. (Bryant et al.). The eight manuscripts reported one of the largest cohorts of patients treated with online MRgRT of liver metastases focusing on oncological outcome, toxicity, patient-reported outcome measures (PROMs), and quality of life (Uder et al.). The ninth manuscript analyzed the role of MRgRT as a potential to become a widely utilized treatment platform and transform the radiation oncology treatment process just as earlier disruptive radiation therapy technologies have done (Ng et al.). Tenth manuscript, aimed to optimize patient selection for stereotactic ablative radiotherapy in patients with locally advanced pancreatic cancer after initial chemotherapy (Doppenberg et al.). The eleventh manuscript reported the outcome and toxicity of the first 200 patients with prostate cancer treated with MRI-Linac (Pridan et al.).

This editorial highlights the implications of recent research findings in this breakthrough technology. This Research Topic of the journal guide Physicians and Medical Physicists who are starting their voyage with this new technology and speed up their learning curve time in their new journey.

## Author contributions

All authors listed have made a substantial, direct, and intellectual contribution to the work and approved it for publication.
